# Carvedilol, Bisoprolol, and Metoprolol Use in Patients With Coexistent Heart Failure and Chronic Obstructive Pulmonary Disease: Erratum

**DOI:** 10.1097/01.md.0000494747.37187.c1

**Published:** 2016-08-26

In the article “Carvedilol, Bisoprolol, and Metoprolol Use in Patients With Coexistent Heart Failure and Chronic Obstructive Pulmonary Disease”,^1^ which appeared in Volume 95, Issue 5 of *Medicine*, an acknowledgment was originally omitted. The article has since been corrected online.
